# Evaluating the Necessity of Distal Tibia-Fibular Syndysmosis Screw Fixation in the Gradual Correction of Angular Lower-Limb Deformities

**DOI:** 10.7759/cureus.89667

**Published:** 2025-08-09

**Authors:** Demah M Benfaris, Khaled AlJedia, Abdulaziz Shadid, Hamza M Alrabai

**Affiliations:** 1 Orthopedics, College of Medicine, King Saud University, Riyadh, SAU; 2 Family Medicine, King Fahad Medical City, Riyadh, SAU

**Keywords:** coronal limb alignment, fixation of syndesmosis, limb deformity, limb deformity correction, syndesmosis screw, taylor-spatial-frame, varus angulation

## Abstract

Background: Gradual correction of lower-limb angular deformities using external fixators such as the Taylor Spatial Frame (TSF) is a well-established technique for addressing complex, multiplanar deformities. A common yet understudied adjunct to this method is the use of a distal tibio-fibular syndesmotic screw to stabilize the ankle mortise during correction. Despite being frequently practiced, the necessity and efficacy of this intervention remain unclear.

Objective: To evaluate the clinical and radiographic outcomes of patients who underwent gradual correction of lower limb deformities using TSF without the placement of a distal tibio-fibular syndesmotic screw.

Methods: A retrospective review was conducted on a sample of 20 limb-deformity-correction procedures performed between September 2017 and January 2020 at a single tertiary care center. Radiographic parameters, including medial clear space (MCS), tibio-fibular clear space (TFCS), and talocrural angle (TCA), were measured pre- and post-operatively. Functional outcomes were assessed using the Foot and Ankle Disability Index (FADI). Statistical analysis was conducted using paired t-tests, Wilcoxon signed-rank tests, Mann-Whitney U tests, and Chi-square tests.

Results: No statistically significant differences were observed in MCS or TFCS following surgery. TCA improved significantly (p=0.009), indicating successful correction of angular deformity. However, there was no significant difference in FADI scores between patients who had syndesmotic screw placement and those who did not (p=0.523). Furthermore, FADI scores for post-operative patients were lower than those of the normative population (p<0.001).

Conclusion: The use of a distal tibio-fibular syndesmotic screw during TSF-assisted gradual correction of lower-limb angular deformities does not significantly influence radiographic or functional outcomes. Given the additional operative time and resources involved, its routine use should be reconsidered. Larger, prospective studies are warranted to establish definitive clinical guidelines.

## Introduction

Unlike deformities of the upper extremities, pathologic lower-extremity angular deformities can pose significant pain and discomfort, and alter the quality of life for patients who experience them. Various conditions have been linked to the development of lower-limb angular deformities, including metabolic conditions, such as rickets or renal osteodystrophy; post-traumatic changes, whether it’s malunion or partial damage to the growth plate; neoplastic conditions; and developmental conditions, namely, Blount’s disease; amongst others. Regardless of the etiology, this condition can be devastating for the patient. Resulting malalignment can be in the coronal plane; valgus or varus, sagittal plane; procurvatum or recurvatum or axial plane with rotational malalignment. Regardless of etiology, patients typically present with multiplanar deformity [1-3].

The choice of treatment varies depending on age, maturity, weight, subtype, stage, and severity. Operative measures extend to include the less-invasive hemi-epiphysiodesis to epiphysiolysis and ultimately to osteotomies with either acute correction or gradual correction with the aid of an external device. In the case of a multiplanar deformity, in Blount’s disease, for instance, most treatment measures target the varus malalignment with little to no correction of the other planar deformities. However, osteotomy with gradual correction using external fixators accommodates multiple planes of correction with respect to dynamic compression and the advantage of early weight bearing as well as lengthening, making it one of the most common approaches used in managing lower limb angular deformities [4-8].

The efficacy of this procedure extends beyond the scope of Blount's disease to be of use in other pathologic or even traumatic causes of malalignment in children and adolescents, whether it is varus or valgus. Osteotomy with gradual correction has been used to treat malalignment secondary to multiple hereditary exostoses for instance [3,9].

Gradual correction of the axis is achieved by placing circular or monolateral external fixators, the former being more popular for its versatility. The Ilizarov apparatus and the Taylor Spatial Frame (TSF) are widely utilized circular external fixators [6,7,10]. In the gradual correction of angular deformities distal to the knee joint, placing a temporary tibio-fibular syndesmotic screw to ensure that the tibio-peroneal mortise is not altered during correction is common practice. However, this technique is not without potential risks, including hardware-related irritation, screw breakage, syndesmotic over-constraining, and the need for subsequent removal. We intend to test the necessity of this practice, as there is no clear evidence for its efficacy as a mainstay of treatment. 

## Materials and methods

Research design and procedure

After obtaining the approval of the institution’s ethics review board and in accordance with international research ethics standards, we searched the hospital records for patients who underwent osteotomy with gradual correction using TSF without placing a distal tibio-fibular syndesmotic screw between the period of September 2017 and January 2020 at King Khalid University Hospital in Riyadh, Saudi Arabia. All patients who underwent correction using osteotomy and TSF application for gradual correction were considered; adolescents and adults. They were all operated on by one of two experienced reconstructive surgeons (X, D), using the same technique. 

Demographic and pre-operative clinical data was retrieved, reviewed and analyzed. Sagittal and coronal plane deformities were mainly evaluated using standing whole-limb plain radiography, while rotational deformity was determined by clinical assessment by the surgeon himself (X, D). Pre-operative and post-operative radiographic measurements were made by two independent investigators (Y, Z) using our institution’s software system (Centricity, GE Healthcare, USA). Where conflict in taking measurements arose, investigators turned to X for resolution.

Investigators measured several key radiographic parameters to evaluate deformity and correction outcomes. The medial proximal tibial angle (MPTA) was defined as the angle subtended medially between the horizontal line of the tibial plateau and a vertical line extending from the center of the knee to the center of the ankle, as visualized on a true anteroposterior (AP) radiographic view. The posterior proximal tibial angle (PPTA) was determined as the posterior angle between a horizontal line along the proximal tibial joint line and a longitudinal line passing through the center of the knee to the center of the ankle on the lateral radiograph. Tibial length was measured in centimeters as the linear distance between the center of the proximal tibia and the center of the distal tibia on the AP view.

The lateral distal tibial angle (LDTA) was defined as the lateral angle formed between a longitudinal line extending from the proximal to distal tibial centers and a transverse line along the distal tibial joint surface on the AP radiograph. The anterior distal tibial angle (ADTA) represented the anterior angle formed between a similar longitudinal axis and the distal tibial joint line, but assessed using a lateral view radiograph. The medial clear space (MCS) was measured in millimeters as the distance between the lateral surface of the medial malleolus and the medial surface of the talus on the AP radiograph. Additionally, the tibio-fibular clear space (TFCS) was assessed as the millimeter distance between the incisura fibularis and the medial cortex of the fibula, specifically at 1 cm above the distal tibial end. Finally, the talocrural angle (TCA) was calculated as the angle formed between the horizontal line of the distal tibia and a line connecting the inferior ends of the malleoli.

To assess functional outcomes, the Foot and Ankle Disability Index (FADI) was administered post-operatively. Investigators (Y, Z) conducted telephone interviews with patients to complete the questionnaire as part of the follow-up assessment [8].

Inclusion and exclusion criteria

Patients were included in the study if they were adolescents or adults (aged 12 years and above) who underwent proximal tibial osteotomy with gradual correction using the TSF without placement of a distal tibio-fibular syndesmotic screw, had available pre- and post-operative standing full-length radiographs, and had a minimum follow-up period of 12 months. Patients were excluded if they had a history of ankle or distal tibial fractures, pre-existing ankle joint pathology such as severe arthritis or congenital deformities, incomplete clinical or radiographic records, or if a syndesmotic screw was used during the correction procedure.

Statistical analysis

Statistical analysis was performed with SPSS version 21. The normality of data was tested by the Shapiro-Wilk test. Differences between the two groups were calculated using t-test, Mann-Whitney U test, and Wilcoxon signed-rank test. A Chi-square repeated measure was conducted to determine whether there was a statistically significant difference between the use of a syndesmotic screw in correction as opposed to correction without a syndesmotic screw for improving several aforementioned radiographic parameters. P<0.05 was considered as significant.

## Results

Our findings are based on 20 gradual malalignment procedures, 8 (42.1%) of which were performed on the right side and 11 (57.9%) were on the left. 16 (80%) patients did not have a syndesmotic screw placed, whereas 4 (20%) did. In our population, 50% of participants were male. Mean age was 29.3 with an SD of 14.6. Pre- and post-operative radiographic parameters as described in Table 1.

**Table 1:** Pre- and post-operative radiographic parameters *: p<0.05; **: p<0.01 MCS: Medial clear space; TFCS: Tibio-fibular clear space; TCA: Talocrural angle

Measurement	Pre-operative				Post-operative	
	MCS	TFCS	TCA	MCS	TFCS	TCA
Mean	3.31 mm	5.48 mm	77.39	3.46 mm	4.71 mm	82.27
SD	0.79 mm	1.83 mm	6.11	1.06 mm	1.6 mm	9.65
Minimum	1.7 mm	2.00 mm	66.80	1.3 mm	1.4 mm	43.00
Maximum	4.6 mm	9.9 mm	87.00	5.5 mm	7.5 mm	88.70
P value	0.412	0.031*	0.008**			

A Shapiro-Wilk test was conducted to determine the normality of distribution among the following data: MCS, TFCS, and TCA pre- and post-operative. In addition to FADI scores. All tested variables were normally distributed, except for post-operative TCA, and FADI scores. 

FADI score between normal population and post-operative patients. A Mann-Whitney U test revealed that FADI scores of the normal population (M=99.5) were significantly higher than the post-operative patients group (M=85.12), p<0.001 (Table 2).

**Table 2:** FADI scores (post-operative and normal population) FADI: Foot and Ankle Disability Index

Measurement	FADI scores
Mean	94.7059
SD	11.04926
Minimum	55.80
Maximum	100.00
P value	0.274

To assess the impact of syndesmotic screw placement on post-operative TCA, a Chi-Square test was conducted after excluding patients who had normal TCA values (between 79 and 87) pre-operatively. The results indicated no statistically significant association between syndesmotic screw placement and post-operative TCA, χ²(1, N=10)=1.33, p=0.25, Cramér’s V=0.36, suggesting a small-to-moderate effect size. When comparing FADI scores between patients with and without syndesmotic screws, a Mann-Whitney U test revealed no statistically significant difference between the two groups. Patients with syndesmotic screws had a mean FADI score of 92.3, while those without screws had a mean of 83.5 (p=0.523). For the MCS, a paired sample t-test showed that the mean pre-operative MCS (M=3.31, SD=0.79) was not significantly different from the post-operative MCS (M=3.46, SD=1.06), with a p value of 0.592, indicating no meaningful change following surgery. Similarly, analysis of the TFCS using a paired sample t-test demonstrated no statistically significant difference between the pre-operative value (M=5.48, SD=1.83) and the post-operative value (M=4.71, SD=1.6), with p=0.105. In contrast, evaluation of the TCA using a Wilcoxon signed-rank test revealed a statistically significant improvement following surgery. The pre-operative TCA (M=77.39) was significantly lower than the post-operative TCA (M=82), with a p value of 0.009, suggesting that the gradual correction technique effectively addressed angular deformity. To assess the impact of syndesmotic screw placement on post-operative TCA, a Chi-square test was conducted after excluding patients who had normal TCA values (79-87) prior to surgery. The test revealed no statistically significant association between screw placement and post-operative TCA (p=0.25) (Table 3).

**Table 3:** Summary of data MCS: Medial clear space; TFCS: Tibio-fibular clear space; TCA: Talocrural angle; FADI: Foot and Ankle Disability Index; L: Left; R: Right; NA: Not available

	Diagnosis	Side	Syndesmotic screw	Sex	Age	MCS		TFCS		TCA		FADI
						Pre-operative	Post-operative	Pre-operative	Post-operative	Pre-operative	Post-operative	
1	Blount’s Disease	L	No	M	48	3.3 mm	4.7 mm	7.3 mm	4.4 mm	82.2	85	100
2	Post-Traumatic	L	No	M	22	3.5 mm	1.3 mm	5.2 mm	5.4 mm	69.5	87.9	100
3	Blount’s Disease	R	No	F	22	2.6 mm	3.3 mm	4.8 mm	4.2 mm	73.5	81.5	76
4	Blount’s Disease	L	No	M	28	2.7 mm	3.4 mm	6.4 mm	5.3 mm	72.1	81	92.3
5	Blount’s Disease	L	No	M	28	2.2 mm	2 mm	5.8 mm	3.9 mm	66.8	85.5	65.4
6	Multiple Hereditary Exostoses	L	No	F	19	4 mm	5.3 mm	0 mm	0 mm	78	88.3	82.7
7	Blount’s Disease		No	M	31	3 mm	3.3 mm	9.9 mm	5.3 mm	76	85.2	62.5
8	Blount’s Disease	R	No	F	8	3.4 mm	3.7 mm	0 mm	0 mm	84.3	85.6	55.8
9	Post-Traumatic	L	No	F	53	1.7 mm	3.3 mm	2.3 mm	4.1 mm	73.8	79.3	87.5
10	Blount’s Disease	L	Yes	F	13	2.7 mm	3.3 mm	2 mm	2 mm	85.6	85.6	NA
11	Rickets	R	Yes	F	15	4.2 mm	1.9 mm	5.5 mm	3.5 mm	80	83.1	90.4
12	Blount’s Disease	R	Yes	F	15	2.6 mm	3.6 mm	5.3 mm	6.6 mm	85.5	88.7	100
13	Blount’s Disease	R	Yes	M	18	3.8 mm	3.3 mm	5.3 mm	1.4 mm	75.6	43	86.5
14	Post-Traumatic	L	No	F	53	3 mm	5.5 mm	5.6 mm	4.1 mm	74.3	87.3	87.5
15	Blount’s Disease	L	No	F	25	4.5 mm	0.47 mm	0.59 mm	0.34 mm	76.7	81.2	98.1
16	Blount’s Disease	R	No	M	28	3.8 mm	0.41 mm	0.57 mm	0.62 mm	75.8	83.9	100
17	Blount’s Disease	R	No	M	25	4.4 mm	0.35 mm	0.38 mm	0.48 mm	83.5	81.6	NA
18	Blount’s Disease	R	No	M	25	4.6 mm	0.3 mm	0.68 mm	0.75mm	80.8	85.5	62.5
19	Blount’s Disease	L	No	M	30	3.2 mm	0.35 mm	0.73 mm	0.67 mm	87	80.1	NA
20	Blount’s Disease	L	No	F	14	3 mm	0.25 mm	0.39 mm	0.6 mm	66.9	86.2	100

## Discussion

Gradual correction of angular lower-limb deformities has been widely used and described in the literature. To the best of our knowledge, there has been no emphasis on the efficacy of placing a distal tibio-fibular syndesmotic screw during the procedure. However, multiple studies have mentioned its use as part of their surgical technique without stating the validity of such an approach [5,11,12]. In fact, we encountered numerous studies that focused on reconstructive strategies in managing angular deformities without clearly mentioning the placement of a syndesmotic screw in their surgical technique, only to find that they indeed utilized syndesmotic screws as evident on radiographs [13,14].

In a review by De Pablos et al., the use of a syndesmotic screw was recommended to ensure that the ankle mortise is not altered during correction [5]. Additionally, some literature suggests that placement of a syndesmotic screw may be considered when deformity correction results in substantial lengthening, such as over 2.5 cm, to protect against proximal fibular migration [13].

Interestingly, a comparative study that looked into the effectiveness of fibular osteotomies in gradual correction mentioned that the standard protocol not only involves osteotomy of the fibula but also fixing it to the tibia at least distally using a screw or Ilizarov wires. The authors did not fix the fibula for their no fibular osteotomy group and found that there was no significant difference between the two groups. However, this study emphasized fibular osteotomy rather than distal tibio-fibular stabilization [14].

Moreover, in the TSF manual on correction of tibial deformities, stabilization of the proximal and distal syndesmosis with a screw or wire is suggested for the prevention of dislocation in cases with "pronounced" angular deformity or in cases where a fibular osteotomy in distraction is performed [15].

The true scientific rationale behind placement of a distal tibio-fibular syndesmotic screw or wire remains vague in the literature. To the best of our knowledge, reliable randomized controlled trials that answer this question are non-existent. In spite of this, distal tibio-fibular stabilization is classically taught in the gradual correction of angular deformities. We believe this intervention is time-consuming, increases operative burden, and is not proven to be cost-effective. Based on our findings, there is no significant difference in post-operative functional outcomes when comparing patients who received distal tibio-fibular stabilization to those who did not.

These findings may influence surgical decision-making by encouraging surgeons to reconsider the routine use of distal tibio-fibular screws in cases where there is no strong indication, such as minimal lengthening or absence of fibular osteotomy. Avoiding unnecessary stabilization may reduce operative time, hardware-related complications, and overall costs, without compromising patient outcomes.

Limitations to this study include its retrospective design, small sample size, and being conducted at a single center. Further stronger evidence, preferably in the form of prospective randomized studies, is suggested to reach a scientific consensus.

## Conclusions

Based on our retrospective analysis, the use of a distal tibio-fibular syndesmotic screw during gradual correction of angular lower-limb deformities did not result in significantly improved radiographic or functional outcomes. These findings suggest that routine syndesmotic screw placement may be unnecessary, particularly in cases without significant lengthening or fibular osteotomy. Future prospective studies with larger sample sizes are warranted to confirm these findings and guide evidence-based surgical decision-making. 
